# Case report: Be alert to herpes zoster after total knee arthroplasty

**DOI:** 10.3389/fsurg.2023.1042482

**Published:** 2023-05-05

**Authors:** Zhanqi Wei, Xisheng Weng

**Affiliations:** ^1^ Department of Orthopaedics, Peking Union Medical College Hospital, Chinese Academy of Medical Sciences and Peking Union Medical College, Beijing, China; ^2^ School of Medicine, Tsinghua University, Beijing, China; ^3^ State Key Laboratory of Complex Severe and Rare Diseases, Peking Union Medical College Hospital, Chinese Academy of Medical Sciences and Peking Union Medical College, Beijing, China

**Keywords:** herpes zoster (HZ), varicella zoster virus (VZV), total knee arthroplasty (TKA), infection, pain

## Abstract

An 88-year-old woman started complaining of severe pain at the right knee and above at three weeks after right total knee arthroplasty (TKA). The empirical treatment cannot effectively control the progress of pain. The lesion was eventually diagnosed to be due to herpes zoster (HZ). The finding of HZ was unexpected in this case, because HZ is extremely rare in patients after TKA.

## Introduction

Herpes zoster (HZ) is a disease caused by reactivation of varicella zoster virus (VZV). The reactivation of VZV may occur spontaneously or in the context of impaired cell-mediated immunity (1). However, HZ is extremely rare in patients undergoing total knee arthroplasty (TKA). In this article, we present the case of an old female patient who developed HZ after TKA.

## Case report

An 88-year-old woman was admitted to the hospital for right TKA. She had previously undergone left TKA six months ago. She had not received glucocorticoid pulse therapy.

**Figure 1:**
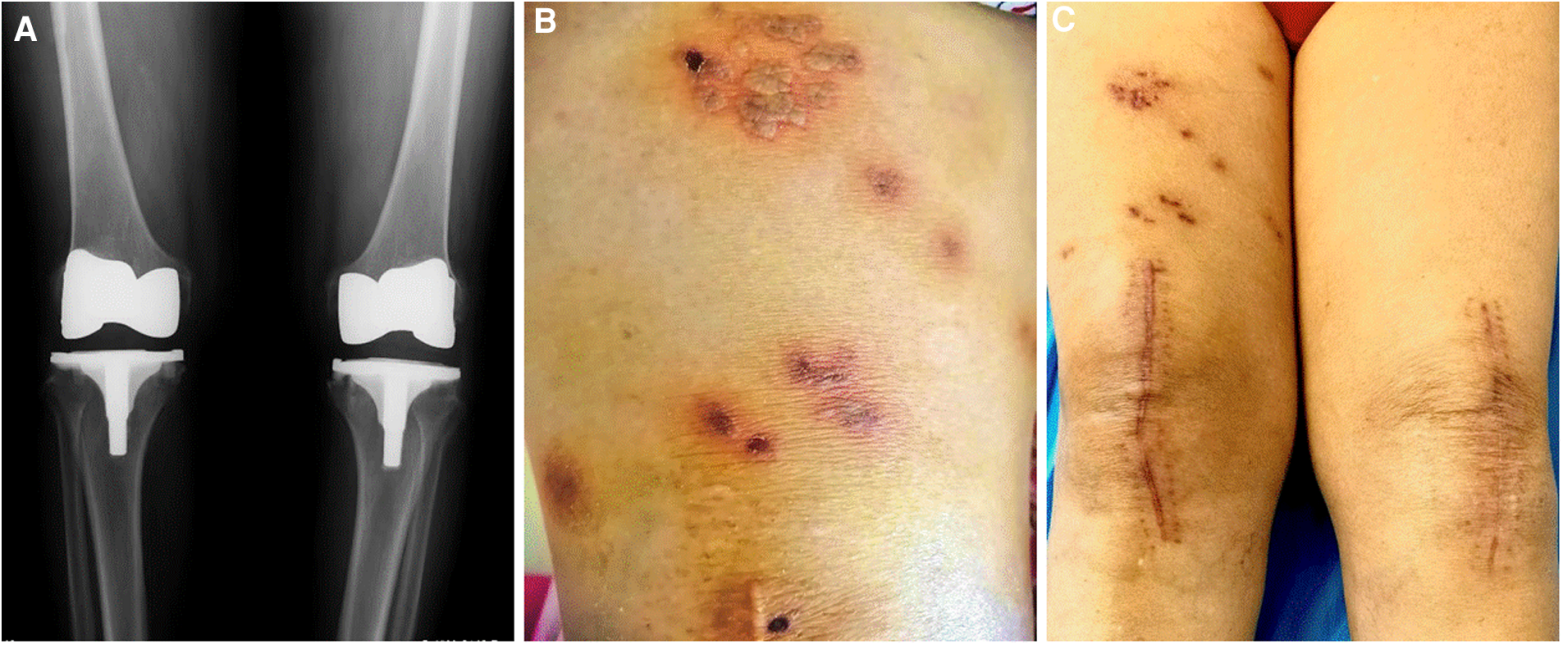
X-ray and macroscopic imaging of the patient. (**A**) Anteroposterior radiographs of dual knees. (**B**) Macroscopic image of characteristic vesicles. (**C**) Macroscopic image of the lesion after treatment.

After right TKA, the patient presented in pain around the wound (VAS 4–5), with no blood or fluid leakage. ESR: 22 mm/h (preoperative)→58 mm/h (postoperative day 3), hsCRP: 1.87 mg/L→86.50 mg/L, albumin 39 g/L→34 g/L, transferrin 2.6 g/L→2.8 g/L. The patient remains well with no fever. She was administered routine postoperative analgesia, antiinfective and anticoagulant therapy. She claimed that the pain gradually eased. On the fifth day after TKA, the x-ray showed no loosening of the prosthesis and no significant fractures around the prosthesis (Figure 1A), and the patient was then allowed home.

At approximately three weeks after TKA, the patient started complaining of paroxysmal stabbing pain (VAS 5–6) at the right knee and above. She had no fever, and her right knee had normal flexion and extension. The wound appeared healthy, and there were no signs of inflammation. The patient could not present to our hospital due to the lockdown induced by the spread of COVID-19. Thus, we empirically prescribed Dynastat (an analgesic agent) over the phone for the patient. The condition did not improve, and she claimed that the pain had significantly aggravated to become unbearable (VAS 7–8).

Five days after severe and persistent pain, painful erythematous grouped vesicles were observed above the right knee (Figure 1B). The patient was referred to the dermatology department. The lesion was diagnosed to be due to herpes zoster (HZ) according to the clinical symptoms and signs and a Tzanck smear test. She was prescribed Aciclovir (an antiviral agent), Mecobalamin (a neurotrophic agent), and Paracetamol (an analgesic agent). The pain gradually subsided and vesicles started to crust after 14 treatment days. Three months later, an outpatient review showed that the pain had completely disappeared, but there was still marked pigmentation, scarring, and occasional tingling above the right knee (Figure 1C).

## Discussion

HZ is a clinical manifestation of reactivation of VZV that harbours in the dorsal root ganglia after a past primary infection producing chickenpox. The reactivation of VZV is largely attributed to decreased cell-mediated immune function. The finding of HZ was unexpected in our case. The reactivation is suspected to be associated with multiple invasive surgeries in the short term for elderly people with the decline in immune functions, because advanced age and immunosuppression were regarded as predisposing factors for HZ. In addition, the patient claimed to have HZ on her right shoulder 25 years ago. Clinical observations found that about 3.9% of patients experienced a HZ recurrence (2), and the immunocompromised patients had a 25% higher risk of HZ recurrence (3).Pain symptoms around the wound lasting one to two weeks after TKA are very common. Orthopedic surgeons generally consider that the occurrence of these pains is related to surgery. Even if there is long-term pain around the incision, it is difficult to make an accurate diagnosis in time because shingles often appear in the chest and are less likely to appear on the thigh (<2%), and characteristic vesicles always appears later than the pain (4). Therefore, we need to consider the possibility of HZ when patients experience severe and unrelieved pain around the wound after TKA, seeking the help of dermatologists to evaluate the lesions, and immediately take targeted antiviral and neurotrophic treatment.

## Data Availability

The original contributions presented in the study are included in the article/Supplementary Material, further inquiries can be directed to the corresponding author/s.
